# Emissions Control Scenarios for Transport in Greater Cairo

**DOI:** 10.3390/toxics9110285

**Published:** 2021-11-01

**Authors:** Rana Alaa Abbass, Prashant Kumar, Ahmed El-Gendy

**Affiliations:** 1 Global Centre for Clean Air Research (GCARE), Department of Civil and Environmental Engineering, Faculty of Engineering and Physical Sciences, University of Surrey, Guildford GU2 7XH, Surrey, UK; r.moustafa@surrey.ac.uk; 2 Department of Civil, Structural & Environmental Engineering, Trinity College Dublin, D02 PN40 Dublin, Ireland; 3 School of Architecture, Southeast University, Nanjing 210096, China; 4 Department of Construction Engineering, School of Sciences and Engineering, The American University in Cairo, New Cairo 11835, Egypt; ahmed.elgendy@aucegypt.edu

**Keywords:** greater Cairo, transport emissions, emission control scenarios, IVE model

## Abstract

Air pollution is a major cause of premature death in Greater Cairo, but studies on emission control are limited. We used local and international data to predict the impact of transport emission control measures on sector parameters including congestion. The International Vehicle Emission model accordingly estimated quantities of criteria, toxic and global warming emissions produced by on-road vehicles. Emissions were estimated for 2019 base case (2019-BC) and projected for 2030 under the ‘do nothing’ scenario (2030-DNS) and five scenarios: fuel subsidy removal (2030-FSR), road expansions (2030-RE), public transport improvements (2030-PTI), inspection and maintenance (I/M) programs (2030-I/MP), and fuel enhancements (2030-FE). The 2030-FSR would reduce emissions by 11.2% versus 2030-DNS. The 2030-RE resulted in an average increase of 37% in emissions compared with 2030-DNS since it induces more traffic. The 2030-PTI provides alternatives to car travel; hence, cars result in an average drop of 32.8% for all emission types compared with 2030-DNS. The 2030-I/MP exhibited reductions in PM_10_ and toxic pollutants, of 35–54.8% compared with 2030-DNS. The 2030-FE reduced SO_x_, benzene and N_2_O emissions by 91.8%, 81% and 39.1%, respectively, compared with 2030-DNS. The 2030-I/MP is most effective in reducing health damaging pollutants while 2030-PTI positively impacts commuters’ lifestyle.

## 1. Introduction

Air pollution is one of the top five risk factors for disease and premature death in Egypt [1]. In Greater Cairo, the transport sector accounts for 26% of total emissions of particulate matter with a diameter ≤ 10 µm (PM_10_), more than 90% of carbon monoxide (CO), 90% of hydrocarbons, 22% of sulfur oxides (SO_x_), and 50% of nitrogen oxides (NO_x_) [2,3]. Additionally, on-road traffic congestion results in economic losses of up to 4% of the national GDP [4], owing to poor public transit, high age vehicles, and overcrowding [5]. Moreover, rapid urban growth and increased transport demand continue to pressure the system [6,7]. Traffic commuters are direct receptors of on-road transport pollution and are, hence, susceptible to acute and chronic diseases [8,9,10]. Consequently, emission control measures are adopted globally to reduce national health burdens and associated economic losses. Control measures are also targeted at reducing global warming pollutants to protect the livelihoods of urban dwellers [11,12]. Similarly, in Greater Cairo, emission control programs are continuously implemented to address the standing issue of on-road transport pollution [13]. Nevertheless, there has not been a systematic study that assesses the effectiveness of such efforts.

Over the years, Egypt has implemented projects to mitigate on-road transport emissions including banning leaded gasoline, replacing old taxis, strict Inspection and Maintenance (I/M) programs, promoting compressed natural gas (CNG), and improving public transport and road infrastructure [14,15]. Other globally adopted measures include staggering work start times, vehicle sharing, fuel quality enhancement, setting fuel emission standards, and regulating travel speeds [16,17]. Fuel subsidy removal is one of the main instruments adopted in Egypt to reduce congestion [18,19]. Subsidy removals enacted in Egypt in 2014 caused private transport costs to double and public transport costs to increase by 23%, encouraging a shift towards public transport [20,21]. Furthermore, earlier fuel price increases in Egypt reduced gasoline use by 43% and bus miles by 25% [14]. Road expansions are also heavily adopted in Egypt with the aim to alleviate congestion [15,22], despite their ineffectiveness in reducing traffic in the long run [23,24,25]. In 2018/19, over 2000 road projects were carried out, and another 2000 were completed by 2019/20, most of which are in Greater Cairo [26]. On the other hand, public transport improvements are needed in Greater Cairo, where increased car reliance caused a drop in bus travel by 20% over a period of 10 years, despite low fares [27]. In 2015/16, 90% of Egypt’s public transport budget was spent on three new metro lines, bus rapid transit (BRT) systems, monorails, and new railways in Greater Cairo [5]. However, the impact of such investments on relieving traffic congestion has not yet been realized. I/M programs have not been adopted in Egypt on a large scale despite their importance in identifying highly polluting vehicles for timely repair and retirement [28,29]. For example, in Nepal 10–15% of vehicles that failed emission tests had caused 50% of CO emissions, while in Mexico City, I/M tests helped identify defective catalytic converters in public transport vehicles [29,30]. In Greater Cairo in 2008, about 45,000 vehicles and 4436 buses have been inspected with a pass rate of about 70% and 43%, respectively [14]. Inspection tests also led to the banning of two-stroke engine motorcycles in Egypt since 2007 [31]. Nevertheless, a large-scale, centralized I/M program has not been fully implemented in Greater Cairo. Finally, fuel enhancements have reportedly resulted in low vehicle emissions due to efficient fuel consumption and better engine performance [32]. Egypt has made advances in fuel quality by introducing unleaded gasoline and encouraging diesel-fueled vehicles to convert to gasoline or CNG [33]. Moreover, there is a need to reduce sulfur content in diesel as it is currently far from Euro standards [6,34]. Overall, measures to mitigate on-road transport emissions in Greater Cairo have varied in their effectiveness. Fortunately, local efforts are ongoing where transport projects that aim to relieve congestion and reduce emissions are a main part of the Egypt 2030 vision [35].

Emission control measures need to be assessed in terms of effectiveness, impact, and feasibility. A study carried out in 1996 by the Egyptian government, the United States Environmental Protection Agency (U.S. EPA), and the USAID assessed four emission control scenarios for Cairo, showing that I/M programs had not considerably reduced emissions and that new vehicle standards were effective in reducing NO_x_ and evaporative emissions, while reduced fuel volatility was most effective in reducing hydrocarbon emissions, and, finally, a combination of all instruments was proposed as the best route to adopt [36]. Hamed et al. carried out a World Bank study in 2013 showing that the business-as-usual approach would result in an increase in pulmonary and cardiovascular diseases by 137,000 cases and in respiratory illnesses in children by 11.1 million cases, causing premature mortality to increase by 66,300 deaths [3]. Two mitigation scenarios were then investigated where reducing the sulfur content in fuel resulted in notable reductions in SO_x_ and NO_x,_ while replacing the aging fleet resulted in a drop in PM_10_ [3]. In 2019, another World Bank study showed that fuel subsidy removal and the new metro line reduced PM_10_ by 4% and 3%, respectively [1]. However, no comprehensive academic work was conducted for Greater Cairo that sets a systematic methodology to assess the impact of ongoing and future efforts to reduce on-road transport emissions in the long run.

The International Vehicle Emissions (IVE) modeling tool has been used for cities in developing countries that lack data availability to estimate on-road transport emission quantities [37,38]. IVE uses information on vehicle technologies, driving behaviors, and meteorological conditions to produce a comprehensive emissions’ profile of criteria and toxic and global warming pollutants [39]. Results produced by IVE can assist policy makers in setting the most effective control measures to curb air pollution levels across a city. Table 1 lists studies carried out for cities similar to Greater Cairo that have utilized IVE and other modeling tools to estimate the impact of different control measures on the quantities of on-road transport emissions. IVE has been used for Istanbul, Sao Paulo, and Pune, among other populous cities in developing countries but has not been used for Greater Cairo to date.

This study focused on Greater Cairo as an example of growing megacities in developing countries. We attempted to fill a knowledge gap through first estimating and predicting the impact of five emission control measures on traffic flows in Greater Cairo as well as on the commuting populations’ behaviors and decisions for the year 2030. Then, such information was used as inputs to the IVE modeling tool to compute the impact of such measures on emission quantities. The data needed to produce such traffic trend estimations and projections are not readily available for Greater Cairo; hence, this study combined historical local data with findings from international case studies to accurately run the model (Section 2.2). The design of scenarios (i.e., subsidy removal, road expansions, public transport improvements, I/M programs, and fuel enhancements) was based on national plans and international best practices to ensure the relevance of results (Section 2.3). The study focused on emissions produced by the Greater Cairo on-road vehicle fleet, cars, taxis, buses, trucks, and motorcycles across three road types, arterial, highway, and residential. Emission projections were based on the year 2019, for which a preliminary analysis of pollution drivers was carried out in terms of vehicle types, road types, and driving behaviors (Section 3.1). A comprehensive emission profile was then produced for each scenario and benchmarked against a ‘do nothing’ scenario for 2030 to highlight to policy makers the most effective measures regarding pollution reduction (Section 3.2, Section 3.3, Section 3.4, Section 3.5 and Section 3.6). Finally, a more detailed comparison of scenarios was carried out to weigh the benefits of emission reductions against the associated economic costs to understand the feasibility of implementation (Section 3.7).

## 2. Materials and Methods

### 2.1. Study Area

For this study, Greater Cairo refers to Cairo and Giza governorates. Greater Cairo will be the fifth most populous megacity globally by 2030 [54]. Currently, the city has over 20 million inhabitants (~20% of Egypt’s population) and 4 million on-road vehicles (45% of Egypt’s fleet), out of which 60% are over 10 years old [55,56]. Greater Cairo is characterized by a complex road network (Supplementary Materials Figure S1) of arterial roads, bridges, tunnels, residential streets, and alleys surrounded by a ring road and highways that connect the different zones [7]. The public transport system is characterized by aging fleets, overcrowding, and declining service frequencies [5]. Commuters rely on shared taxis and informal microbuses that make up 83% of motorized trips [2]. The city also has very few designated cycling lanes and pedestrian areas. Geographically, Greater Cairo is bordered by the Mokattam Hills and the Eastern Desert from one side and by the Abu-Rawash Hills and the Western Desert on the other, making it susceptible to dust storms [57]. The city has low annual precipitation (22–29 mm) and high temperatures (18–45 °C). 

### 2.2. Emission Modelling

The IVE model (version 2.0) was jointly developed by researchers at the International Sustainable Systems Research Center and the University of California at Riverside and funded by the U.S. EPA, Office of International Affairs, USA [38]. IVE adopts a bottom-up approach to predict how different transportation management strategies will affect local emissions and measure the progress of reducing emissions over time [44]. IVE uses three critical components to develop accurate mobile source emission inventories: vehicle base emission rates, vehicle activity, and vehicle fleet distribution [43]. The emission estimation process in the IVE model is to multiply the base emission rate (for each technology) by each of the correction factors (defined for each vehicle technology, and are dependent on location-specific parameters such as temperature, relative humidity, and I/M programs) and the amount of vehicle kilometers travelled (VKT) for each technology to produce the total amount of emissions produced [38]. Model inputs include environmental conditions, fuel quality, distances driven, average speeds, number of ‘starts’ (defined as the number of times a vehicle ‘starts’ after the engine has been turned off), vehicle soak patterns (which is the time during which the vehicle is turned off before being started again), AC usage, and vehicle technologies [40]. The model then produces a comprehensive emissions’ profile of criteria pollutants (CO, volatile organic compounds (VOC), evaporative emissions of volatile organic compounds (VOC_evap_), NO_x_, SO_x,_ and PM_10_), toxic pollutants (lead, 1,3-butadiene, acetaldehyde, formaldehyde, ammonia (NH_3_), and benzene), and global warming pollutants (CO_2_, nitrous oxide (N_2_O), and methane (CH_4_)) [38]. IVE input data are grouped into location-specific and fleet-specific parameters, as discussed in Section 2.2.1 and Section 2.2.2.

#### 2.2.1. Location-Specific Parameters

**Figure 1:**
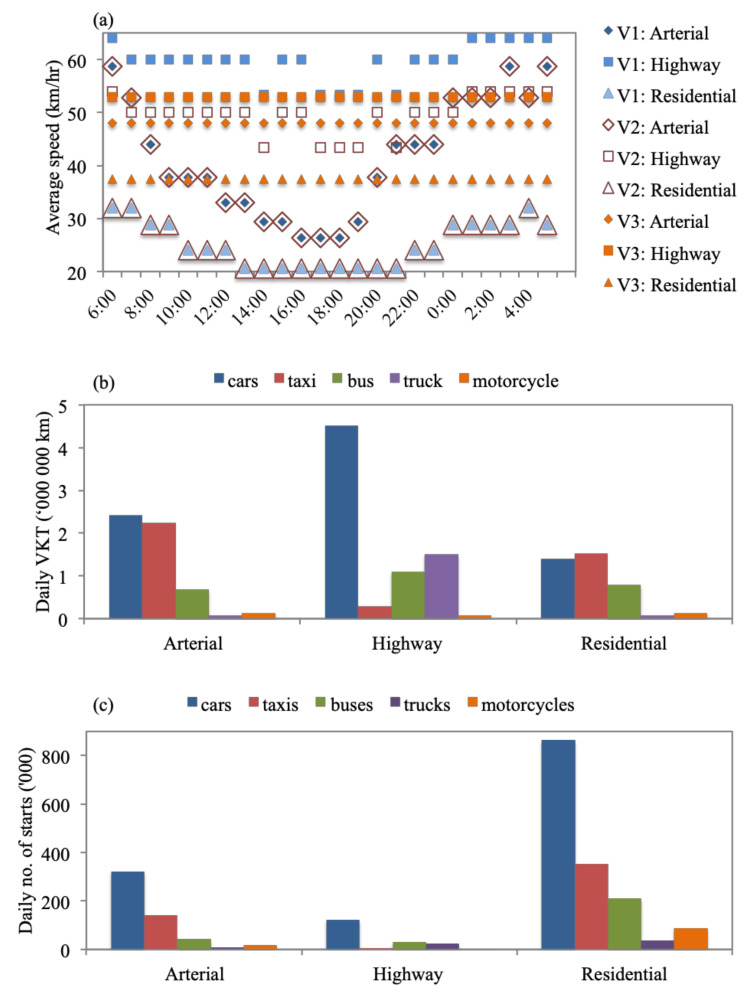
(**a**) Hourly average speed in Greater Cairo where V1 indicates cars and taxis, V2 is buses and trucks, and V3 is motorcycles where each group is considered to have similar driving speeds [59]. (**b**) Daily VKT (distance traveled in km) by each vehicle type across the three studied road types [27,55,56]. (**c**) Daily number of ‘starts’ by each vehicle type across the three studied road types [45,51].

Location-specific parameters included meteorological data, fuel quality, average velocities, distances driven, and driving behaviors. Data availability was a significant challenge for this study [58]. Therefore, we used national reports and literature findings to derive the required parameters. The city is at a latitude and longitude of 30.0444° N and 31.2357° E and an altitude of 23 m [59]. Thursday, 24 October 2019, was chosen to simulate a standard workday in Greater Cairo for the base case scenario, with an average temperature of 28.4 °C and average relative humidity of 55.7% [60]. Lead content in gasoline was set to zero, sulfur and benzene at 600 ppm and 3%, respectively, and oxygenate at 2.5%, while diesel sulfur content was at 5000 ppm [33,34,61]. Five vehicle types were studied, car, taxi, bus, truck, and motorcycle on three road types, arterial, highway, and residential. A Google maps exercise was carried out to derive the hourly speed profiles for each vehicle type on each road type where the time taken to travel a predefined distance was used to calculate the speed at each hour of the day (Figure 1a). The total hourly VKT by the Greater Cairo fleet was derived using Equation (1). For cars, trucks (on residential and arterial roads), and motorcycles, the average distances covered within a trip were used as the hourly VKTs since trips were typically completed within 1 h for these vehicle groups. For taxis, trucks (on highways), and buses, typical vehicle speeds were used as the hourly VKTs since service vehicles were expected to operate continuously (longer than 1 h). The 2019 Greater Cairo vehicle fleet size was used to estimate the total number of vehicles on the street (Figure S2). This study only considered vehicles registered in Greater Cairo as they represent the majority of vehicles traveling within the city on a daily basis. The distribution of vehicles over the three road types and throughout the day was based on field data collected by the World Bank [27]. To simulate a busy weekday, it was assumed that 70% of the fleet drove on arterial roads, 50% on highways, and 80% within residential zones throughout the day. Figure 1b shows the daily VKT, while Figure S3 shows the hourly VKT profiles.

𝑉𝐾𝑇 (𝑘𝑚)= 𝑑𝑖𝑠𝑡𝑎𝑛𝑐𝑒 𝑡𝑟𝑎𝑣𝑒𝑙𝑒𝑑 𝑏𝑦 𝑜𝑛𝑒 𝑣𝑒*h*𝑖𝑐𝑙𝑒 (𝑘𝑚) × 𝑛𝑢𝑚𝑏𝑒𝑟 𝑜𝑓 𝑣𝑒*h*𝑖𝑐𝑙𝑒𝑠 (1)

Driving behaviors are characterized by the number of ‘starts’, vehicle-specific power (VSP), engine stress, and soak patterns. Field data collection of these parameters was beyond the scope of this study; hence, data from similar cities were used to compile the needed data sets. The number of ‘starts’ was derived using the trends observed in Chennai, Kathmandu, Hanoi, Hong Kong, and Pune since these cities have similar congestion levels to Greater Cairo (Figure 1c and Figure S4) [45,51]. VSP, engine stress, and soak pattern distribution data (Section S1) produced by IVE developers for Istanbul were used since they were most similar to Greater Cairo in terms of altitude, congestion levels, fleet composition, average speeds, and road grade [62]. Furthermore, IVE Istanbul data were previously used as a reference for Greater Cairo in a World Bank traffic congestion study [27]. Fifteen location files were prepared for each of the five vehicle types to capture their activity patterns on each of the three road types.

#### 2.2.2. Fleet-Specific Parameters

Fleet-specific parameters included vehicle type, weight, age, fuel type and AC usage, air/fuel control, and exhaust and evaporative controls [38]. For 2019, fuel type distributions varied across the five vehicle types: ~96% of cars and all motorcycles used petrol, while 47%, 71%, and 85% of taxis, buses, and trucks used diesel, respectively [56]. Natural gas was used for 13% of cars, 16% of taxis, and 2% of buses [56]. Vehicle age was defined by IVE as ranges of total mileage (distance traveled by a vehicle throughout its lifetime) such that 42% of cars travelled between 80K km and 161K km, while 60% of taxis, buses, and trucks travelled for more than 161K km, and 70% of motorcycles travelled more than 50 km [31,63]. Weight and vehicle technology parameters were derived based on reported vehicle types and age [56,63]. Table S1 lists the chosen vehicle distributions for the 2019 base case according to IVE indexes. Five fleet files were produced to capture the vehicle technology distributions for cars, taxis, buses, trucks, and motorcycles.

### 2.3. Scenarios

Data in Section 2.2.1 and Section 2.2.2 were collected for 2019 and then projected to the year 2030 under a ‘do nothing’ scenario (DNS). Egypt has a 2030 vision plan that includes several sustainable on-road transport projects and was, hence, chosen as the target year for this study [35]. Parameters were varied for 2030 based on five emission control scenarios to assess their impact on emission quantities. As discussed in Section 1, scenarios were based on the local agenda and international best practices. The description and design of scenarios are as follows.

- 2019 Base Case (2019-BC): 2019 was used as the base case since it has the most recent data set available for Greater Cairo, as discussed in Section 2.2.1 and Section 2.2.2. The 2019-BC was used to produce future projections for the 2030-DNS and other scenarios.
- The 2030 ‘do nothing’ scenario (2030-DNS): It assumes that the fleet size will increase at an annual rate of 5.4% for cars, 0.3% for taxis, 5.7% for buses, 6% for trucks, and 8% for motorcycles (based on historical growth rates) between 2019 and 2030 [56,64]. The average speed was assumed to drop at an annual rate of 2.3% between 2019 and 2030 [65]. The 2030-DNS assumes no emission mitigation measures have been implemented and was, hence, used as a benchmark for the studied scenarios.
- Fuel subsidy removal (2030-FSR): In 2019, fuel prices had already increased by 6 times over 5 years in Egypt [66]. By 2030, fuel prices are expected to increase by 50% to match international fuel prices, i.e., complete removal of subsidies [67]. This is expected to cause a drop in VKT and in the number of ‘starts’ by 8% compared with 2030-DNS, resulting in 3.4% higher average speeds. This estimate was based on a compilation of international experiences where in France, a 10% increase in fuel prices resulted in a 2.8% fall in traffic in the long run [68]; in Greece, fuel price increases (82% for unleaded and 31% for diesel) resulted in a traffic drop of 15.7% over 5 years [69]; and in Australia, a 1% increase in fuel prices resulted in an 0.04% decrease in hourly traffic flow over 7 years [70]. It was also taken into consideration that in high activity zones of Greater Cairo, fuel price increases are not expected to notably reduce traffic due to a lag in the availability of transport alternatives [14]. Furthermore, in the long run, higher fuel prices are expected to encourage the purchase of more fuel-efficient vehicles and improve driving behaviors [71]. Therefore, the vehicle mix was adjusted such that new vehicles (age < 79 km) are expected to be hybrid, lightweight, and fuel-efficient. IVE indexes listed in Supplementary Materials Table S1 were changed for each fleet file to reflect the different scenarios and to reflect the advances in vehicle technologies.
- Road expansions (2030-RE): Studies found that for congested cities, the elasticity between the increase in VKT and the increase in lanes, in the long run, is 1.0 for highways and 0.75 for arterial and residential roads [24,72]. Considering a projected annual increase in road capacity of 3.5% between 2019 and 2030 in Greater Cairo [27], VKT was increased by an annual rate of 3.5% for highways and 2.6% for arterial and residential roads over 2030-DNS between 2019 and 2030. Consequently, the number of ‘starts’ are also expected to increase. However, the average speeds were left the same as 2030-DNS since congestion relief resulting from road expansion projects is expected to be reversed in the long run, according to international experiences (Section 1). This assumption can be considered a worst-case scenario that is likely for Greater Cairo, being the largest, rapidly growing metropolitan area in the Middle East and North Africa [58]. No changes to the vehicle technology mix were made for this scenario.
- Public transport improvements (2030-PTI): This scenario focused on the impact of improved public transport on reducing car reliance. Based on international experiences in Copenhagen [73], Birmingham [74], Los Angeles [75], and Melbourne [76], it was assumed that 30% of commuters who would have bought new cars every year decided to use public transport instead, 50% of whom would use buses. The rest would use the newly developed BRT, monorail, and metro systems. It was estimated that one bus would replace a minimum of 30 cars, achieving congestion relief [77]. This would result in an annual decrease in cars and an increase in buses, consequently affecting the total VKT and number of ‘starts’ for 2030-PTI versus 2030-DNS. Moreover, an annual decrease in average speeds of 0.26% is estimated between 2019 and 2030 versus the 2.3% annual drop in speed assumed for 2030-DNS [65]. The vehicle technology mix is not expected to change.
- I/M programs (2030-I/MP): A loaded tailpipe centralized I/M program for all vehicles was assessed since it has been reported as the most effective I/M program setting in IVE [37]. The 2030-I/MP is expected to result in the replacement of 30% of old taxis, buses, and trucks and 15% of old cars and motorcycles with newer vehicles. Assumptions were based on international experiences, especially in Rio de Janeiro [78] and Nepal [29]. No changes were made to location-specific parameters.
- Fuel enhancements (2030-FE): There are initiatives to produce ultra-low sulfur content 10 ppm diesel [33]; however, for 2030-FE, realistic fuel quality standards were set (given the 11-year timeframe). For gasoline, sulfur content was set at 50 ppm, benzene at 0.5%, and oxygenate at 0%. Diesel sulfur content was set at 500 ppm. Newer diesel-fueled cars, taxis, and buses for 2030-DNS were assumed to have been replaced by CNG for 2030-FE. Trucks and motorcycles stayed the same since they were not part of national plans to be converted to other fuel types.

### 2.4. Data Analysis

The 2019-BC was first analyzed to capture the main emission contributors in terms of vehicle types, road types, and driving behaviors (start-up versus running emissions) (Section 3.1). Percentage distributions for the different emission types were calculated based on emission quantities produced by the different vehicles, on the studied roads, with reference to the total quantities produced in 1 day in 2019. Then the different control scenarios were discussed in reference to 2030-DNS (Section 3.2, Section 3.3, Section 3.4, Section 3.5 and Section 3.6). Data analysis was based on percentage changes rather than absolute emission quantities, to allow for the applicability of results. For example, the percentage reduction in the quantity of CO_2_ produced for 2030-FSR was calculated in reference to the quantity of CO_2_ produced for 2030-DNS. Then, the average reduction/increase in emissions achieved by each scenario versus 2030-DNS was calculated as the average of percentage changes of all 14 emission types (Section 3.2, Section 3.3, Section 3.4, Section 3.5 and Section 3.6). 

Section 3.7 aims to provide a preliminarily understanding of the costs entailed for each scenario versus the benefits of emission reduction. However, an accurate estimation of scenario costs for Greater Cairo requires access to governmental information. On the other hand, the benefits of emission reductions translate into reduced national health burden and associated economic loss [79]. Several studies have estimated and monitored the number of premature deaths caused by air pollution exposure [80,81,82]. However, to derive the health burden, personal exposure concentrations in μg/m^3^ are needed [83,84], while IVE produces the amount of on-road transport pollutants in tons per day. Furthermore, personal exposure concentrations also represent impacts from other sources of pollution, i.e., background pollutant levels resulting from meteorological conditions. Emission quantities estimated by IVE solely represent emissions produced by on-road transport vehicles. Hence, it was not possible to derive the change in the number of premature deaths resulting from adopting each of the five scenarios based on this study’s results.

Nevertheless, it is acceptable to expect that the change in pollutant loads for each scenario versus 2030-DNS would correspond to changes in health burden and economic losses. This is especially true for the commuting population whose exposure to concentrations of on-road traffic air pollutants can be 3–10 times greater than its exposure to background pollutants [85], owing to its proximity to mobile pollutant sources [86]. Health burden studies have focused on the impact of health-damaging pollutants, including PM_2.5_, N_2_O, VOCs, PM_10_, benzene, 1,3-butadiene, CO, lead, NO_2,_ and SO_2_ [79,87,88]. Hence, it would be ideal that a study focuses on evaluating the effectiveness of each scenario in reducing the quantities of health-damaging pollutants. Nevertheless, Section 3.7 serves as a more holistic comparison of scenarios that include economic costs, which highlights the importance of a dedicated study that would further consider feasibility, health, and social aspects. Moreover, the discussions in Section 3.2, Section 3.3, Section 3.4, Section 3.5 and Section 3.6 were based on comparing the average of percentage changes of all emission types for the five scenarios versus 2030-DNS. In Section 3.7, we also compare the percentage change of the total amount of emissions for the five scenarios in comparison to 2030-DNS.

## 3. Results and Discussion

### 3.1. Overview on Emission Quantity Distributions in 2019

**Figure 2:**
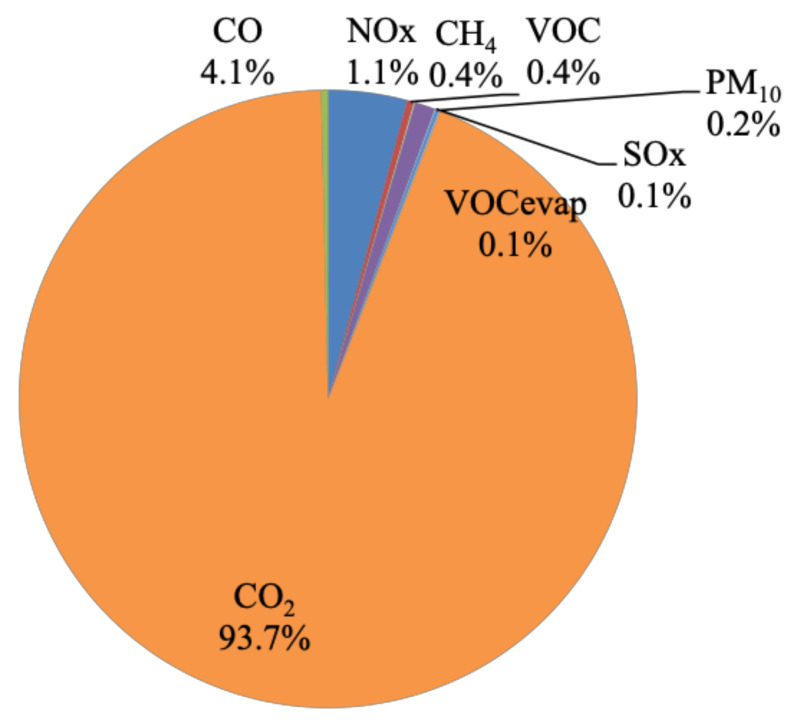
The distribution of different emission types as a percentage of the total quantities produced in 1 day, based in 2019-BC amounts. Toxic pollutants and N_2_O, being negligible (<0.1%), are not labeled on the pie chart.

**Figure 3:**
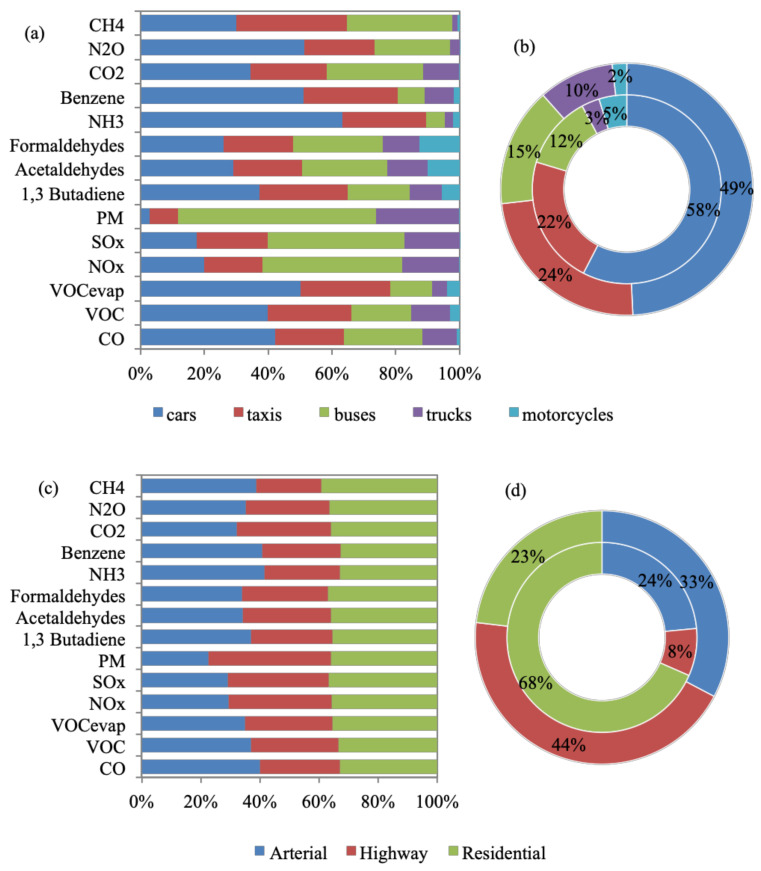
Vehicle type (**a**,**b**) and road type (**c**,**d**) percentage distributions according to (**a**,**c**) 2019 emissions’ quantities; (**b**,**d**) outer donut is VKT and inner donut is number of ‘starts’.

Figure 2 shows that CO_2_ emissions constitute 93.7% of total daily emission quantities, followed by CO (4.1%), NO_x_ (1.1%), CH_4_ (0.4%), VOCs (0.4%), PM_10_ (0.2%), SO_x_ (0.1%), and VOC_evap_ (0.1%). Toxic emissions and N_2_O constitute a negligible percentage (<0.1%) of total emissions. It is evident that on-road transport CO_2_ emissions require national attention. Cars compose 69% of the Greater Cairo fleet (Figure S1), while constituting 49% and 58% of the total daily VKT and number of ‘starts’, respectively (Figure 3a,b). Hence, cars result in 35% of total daily emissions and more than 40% of the daily CO, VOC, VOC_evap_, NH_3_, benzene, and N_2_O emission quantities. Taxis contribute to 24% of total daily emissions, despite making up 3% of the fleet (Figure S1) since they are service vehicles that operate all day (making up 24% of the Greater Cairo daily VKT and 22% of the number of ‘starts’). Furthermore, taxis result in the highest percentage of CH_4_ (35%) among all vehicle types, which could be due to their high vehicle age [89] (Section 2.3). Buses contribute to 44%, 43%, and 62% of NO_x_, SO_x,_ and PM_10_ daily emission quantities, respectively, despite constituting 2% of the fleet. Buses are also high-age service vehicles (covering 15% of daily VKT and 12% of the number of ‘starts’ in the city) (Section 2.3). Furthermore, 71% of buses are diesel-fueled, which could have contributed to the high amounts of NO_x_, SO_x,_ and PM_10_ [90]. Trucks are 81% diesel-fueled and are, hence, the second-highest contributors of daily PM_10_ emissions (26%) [91]. Overall, vehicle age and type of fuel seem to be the most impactful factors affecting pollution quantities despite the fleet size and the VKT per day.

Emission quantities are, on average, equally produced across the three road types, with the least percentages observed on highways (Figure 3c). However, PM_10_ quantities are highest on highways (42%), where trucks spend the largest portion of their time. Figure 3d shows that the largest number of ‘starts’ occurs on residential streets (68%), where most vehicles start and end their trips. VKT distribution is more spread out across all road types, arterial (33%), highway (44%), and residential (23%). Moreover, Figure S5 shows that start-up emissions are, on average, 1.3% of total daily emissions. This shows that start-up emissions do not significantly impact the amount of emissions compared with running emissions. In conclusion, emissions increase during traffic congestion hours, signified by a higher VKT, a higher number of ‘starts’, and lower speeds. 

### 3.2. 2030-FSR

**Figure 4:**
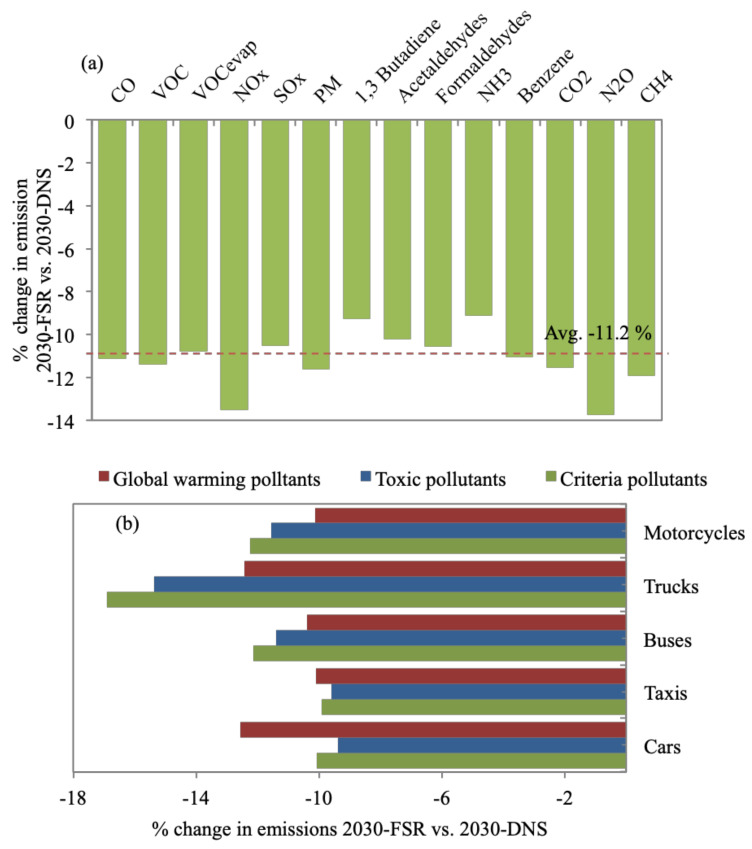
Percent change of (**a**) all daily emission types for 2030-FSR in reference to 2030-DNS and (**b**) provides a breakdown of reduction of each pollutant group for each vehicle type.

Figure 4a shows that 2030-FSR reduces emissions by, on average, 11.2% versus 2030-DNS, owing to reductions in the daily VKT and number of ‘starts’ as well as the use of more fuel-efficient and hybrid vehicles (Section 2.4). The most significant decreases were observed for global warming emissions at an average of 12.4%, followed by criteria emissions at 11.5%, and toxic pollutants at 10.1%, when compared with 2030-DNS. Cars and motorcycles show the highest reduction in global warming emissions versus 2030-DNS, at 12.5% and 12.4%, respectively (Figure 3b). The 2030-FSR assumes that 16% of cars were replaced by lighter weight models with improved air/fuel control and hybrid vehicles, and 7% of motorcycles were replaced with models that have better air/fuel control, while these replacements did not take place for 2030-DNS (Section 2.4). Trucks result in the highest reduction of criteria and toxic pollutants at 15.4% and 16.9%, respectively, when compared with 2030-DNS (Figure 3b). Such reductions might be due to replacing 14% of the 2030 truck fleet with trucks equipped with advanced exhaust control technologies for 2030-FSR (Section 2.4). Overall, 2030-FSR seems to effectively direct drivers’ commuting decisions and vehicle purchases towards contributing to a reduction in emissions.

### 3.3. 2030-RE

**Figure 5:**
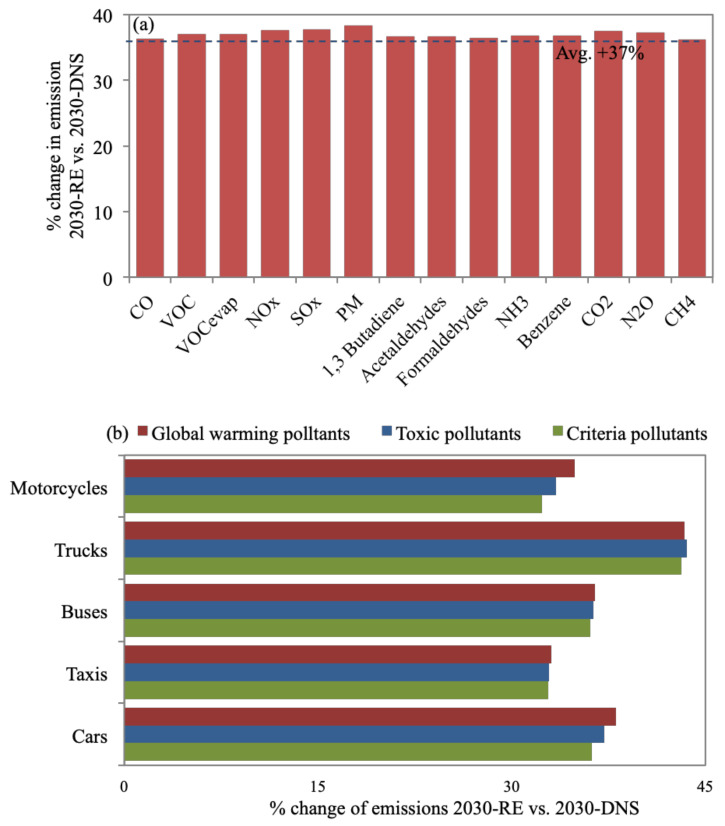
Percent change of (**a**) all daily emission types for 2030-RE in reference to 2030-DNS, while (**b**) provides a breakdown of reduction of each pollutant group for each vehicle type.

Figure 5a shows that 2030-RE resulted in an average increase of 37% in emissions versus 2030-DNS, since the benefits of road expansions are short lived (Section 1). All emission types increase by almost the same percentage since 2030-RE assumes increases in VKT and the number of ‘starts’, with no changes in vehicle technologies. Trucks cause the highest average increase of emissions at 43.6% versus 2030-DNS (Figure 5b) since they travel primarily on highways where most road expansions are planned [72]. Nevertheless, as pointed out in Section 2.3, 2030-RE assumes a worst-case scenario; hence, the increase in emissions might be overstated. It is worth noting that 2030-RE can be beneficial in the short term, where ring roads and bridges provide improved access to suburban areas and, in turn, introduce economic benefits [24]. However, this study looked into long-term impacts on emission levels where the benefits of 2030-RE are reversed and, hence, need to be complemented with other emission reduction measures.

### 3.4. 2030-PTI

**Figure 6:**
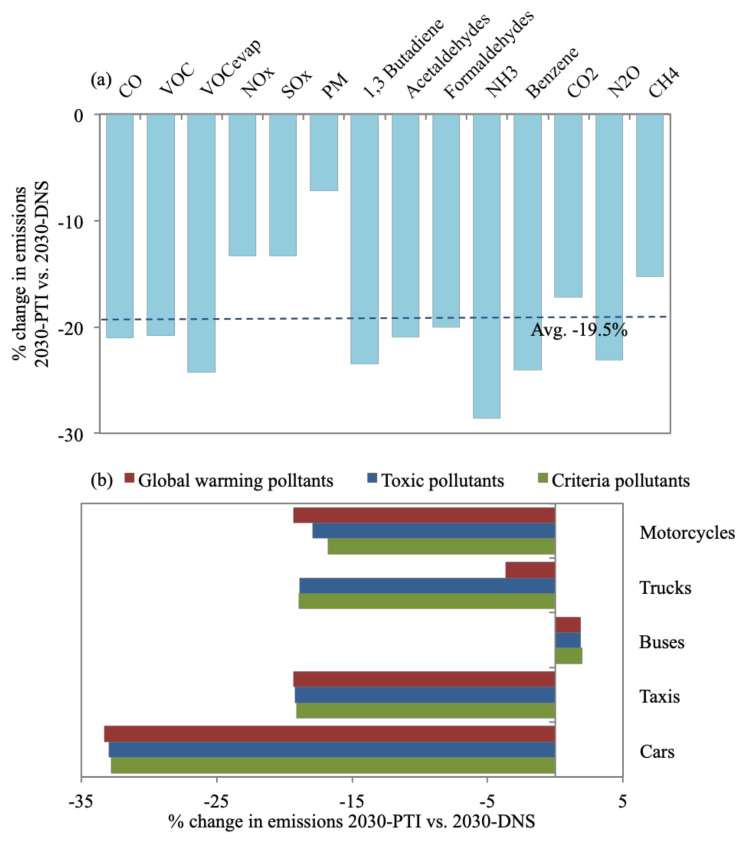
Percent change of (**a**) all daily emission types for 2030-PTI in reference to 2030-DNS, while (**b**) provides a breakdown of reduction of each pollutant group for each vehicle type.

Figure 6a shows that 2030-PTI reduces emissions by, on average, 19.5% when compared with 2030-DNS. The highest average reduction of 28.6% is observed for NH_3_ and the lowest of 7.2% is observed by PM_10_ emissions versus 2030-DNS. Cars result in the most notable reduction for all emission types (average of 32.8%) when referenced to 2030-DNS, as shown in Figure 6b. The 2030-PTI is expected to reduce the number of cars through providing alternatives to car owners (Section 2.4). Emissions from buses increased by, on average, 2.1% versus 2030-DNS since 2030-PTI assumes a rise in the number of buses. However, the increase in bus emissions is minimal and does not affect the overall reduction in emissions. Furthermore, a survey carried out on 460 private car owners in Greater Cairo showed a strong interest in using improved public transport instead of car travel [4]. The 2030-PTI addresses the issue of increased car ownership as a major cause of congestion and would, hence, substantially reduce emissions as well as improve the quality of life in Greater Cairo.

### 3.5. 2030-I/MP

**Figure 7:**
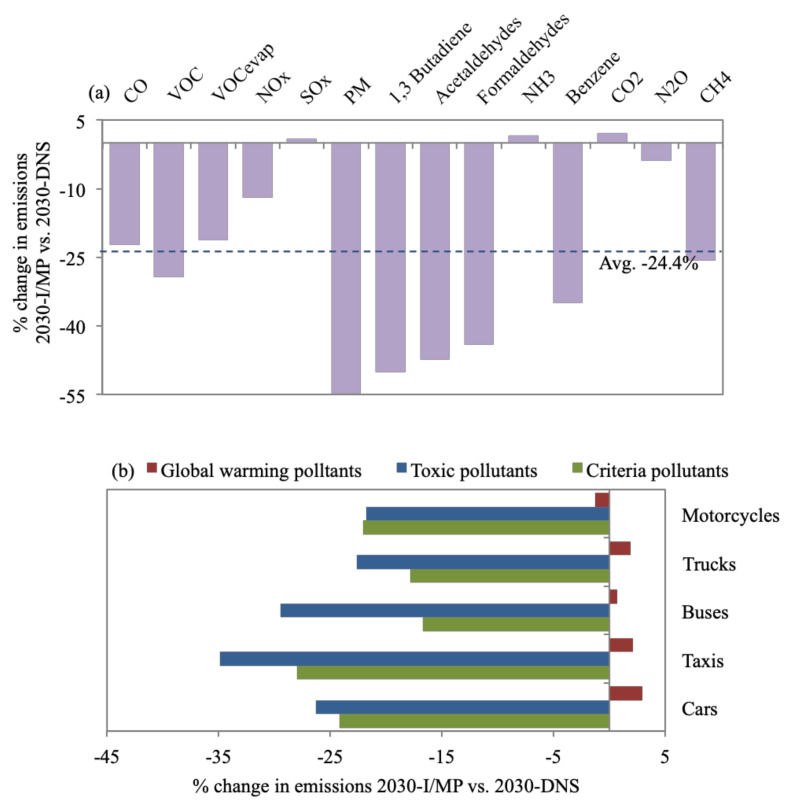
Percent change of (**a**) all daily emission types for 2030-I/MP in reference to 2030-DNS, while (**b**) provides a breakdown of each pollutant group for each vehicle type.

Overall, 2030-I/MP exhibited the most significant average reductions in emissions of 24.4% (Figure 7a) compared with 2030-DNS. Reductions in PM_10_ and toxic pollutants are substantial, ranging between 35–54.8% when benchmarked against 2030-DNS. Figure 7b shows that taxis and buses contribute to the most notable average reductions in emissions of 31.6% and 24.2%, respectively, versus 2030-DNS. Taxis and buses are mostly aging vehicles that would be decommissioned in a timelier manner in the case of 2030-I/MP (Section 2.4). This could indicate that PM_10_ and toxic pollutants are most associated with aging vehicles. However, SO_x_, NH_3,_ and CO_2_ showed a mild increase in emission quantities of 0.9%, 1.7%, and 2.1%, respectively, which could be owed to test emissions [92]. Internationally, I/M programs proved effective in reducing emissions. In California HC emissions dropped by 14–28% [93]. In Beijing, overall emissions dropped by 28–40%. In Canada, HC emissions dropped by 20%, CO by 20%, and NO_x_ by 1% [29]. In Delhi, I/M campaigns that focused on two-wheelers achieved a 39% reduction in CO and 22% drop in HC emission quantities [94]. The 2030-I/MP seems to effectively reduce health-damaging pollutants (PM_10_ and toxic pollutants) since they target the polluting few within the vehicle fleet mix.

### 3.6. 2030-FE

**Figure 8:**
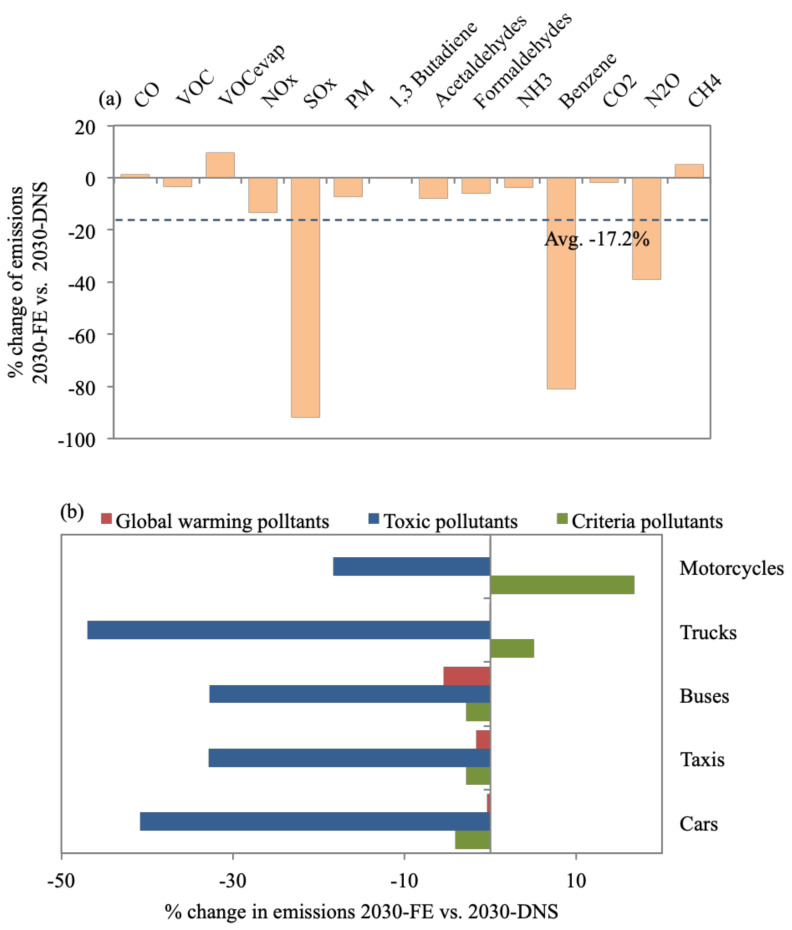
Percent change of (**a**) all daily emission types for 2030-FE in reference to the 2030-DNS, while (**b**) provides a breakdown of reduction of each pollutant group for each vehicle type.

Figure 8a shows an average drop in total emissions of 17.2% for 2030-FE compared with 2030-DNS. SOx, benzene, and N_2_O emissions were reduced by considerable amounts of 91.8%, 81%, and 39.1%, respectively, compared with 2030-DNS. PM_10_ emissions were reduced by 7.4% compared with 2030-DNS, where literature reported a reduction of 25% owing to reducing sulfur content in diesel fuel [95]. In China, the use of gasoline IV reduced PM_2.5_ concentrations by 2.3% and PM_10_ concentrations by 5.1% [96]. Fuels with lower sulfur content resulted in lower emissions of PAHs but did not impact VOCs and aldehydes [97]. This was also the case for 2030-FE as VOCs were reduced by only 3.6% and aldehydes were reduced by on average 8% versus 2030-DNS. However, CO, VOC_evap,_ and CH_4_ emissions increased by 1.2%, 9.5%, and 5.1%, respectively, against 2030-DNS, owing to the conversion of buses to CNG, which has been reported to cause an increase in emissions [98]. In contrast, CO_2_ emissions stayed almost the same, with a mere drop of 2% despite fuel quality improvements. It has been reported that when benzene and aromatic content in gasoline increase, fuel combustion becomes close to ideal, however, resulting in higher CO_2_ emissions [99]. Global warming pollutants also do not seem to benefit from 2030-FE, where minor or no reductions are achieved. Toxic pollutants are the most reduced by 2030-FE, where average emission reductions of 41% and 47% are observed for cars and trucks, respectively, compared with 2030-DNS (Figure 8b). In general, 2030-FE has proven quite effective in reducing certain pollutants, especially SO_x_, benzene, and N_2_O.

### 3.7. Scenarios Comparison

The 2030-FSR requires zero capital investment despite having political ramifications that call for phased implementation [27]. Nevertheless, its non-existent economic cost makes its benefit of reducing criteria, toxic and global warming pollutants, by an average of 12.3%, 11.5%, and 11.1%, respectively, versus 2030-DNS, even more attractive. On the other hand, 2030-RE results in a long-term increase in emissions of on average of 37% over 2030-DNS with associated costs estimated at $US 1 million per mile of new lane/road [27]. This highlights the national costs of 2030-RE besides its negative impact of increased emissions on the long run. Nevertheless, 2030-RE might be beneficial on the short run in relieving traffic as well as providing economic benefits such as improved access to new cities and increased cargo transport routes. The 2030-PTI is expected to reduce emissions by a promising average of 19.5% compared to 2030-DNS; however, it requires massive national investments that can reach US$ 110 million per mile of metro lines, US$ 5 million per mile of BRT system, and US$ 300 million for improved city-wide public transit operations [27]. Nevertheless, 2030-PTI results in an improved quality of life for Greater Cairo inhabitants through job creation, reduced commuting times, and improved road safety and service operating revenues [100] in addition to emission reductions. Hence, the huge cost of 2030-PTI should not discourage its implementation. The highest average reduction of all emission types versus 2030-DNS was observed for 2030-I/MP (24.4%). The costs of an I/M program vary widely according to its participation, identification, and effective repair rates [101]. Hence, it is hard to estimate the funding needed for a city-wide I/M program; therefore, a dedicated study is required in order to estimate its exact cost for Greater Cairo. Nevertheless, a case study in India provides a good reference, where an I/M center was estimated to cost US$ 2.2 million and is expected to generate annual revenue of US$55,000 through service and repair charges, in addition to the economic benefits of job creation [102]. The 2030-FE resulted in a 17.2% average reduction in emission quantities versus 2030-DNS. The 2030-FE entails improving fuel quality and vehicle technologies, whose costs are also hard to estimate. In India, transitioning to ultra-low sulfur fuel was estimated to cost US$ 19 billion and vehicle improvements to cost US$ 170 billion compared to a cumulative benefit of US$ 673 billion [103]. This discussion provided a preliminary understanding of the range of costs entailed for each scenario versus its emission reduction and social benefits.

We also compared the total amount of emissions for each scenario to the total emission quantity for 2030-DNS. The highest reduction versus 2030-DNS was observed for 2030-PTI (17.4%), followed by 2030-FSR (11.5%), while 2030-RE also resulted in an increase in emissions (37.4%). On the other hand, 2030-I/MP resulted in almost no change in the total amount of emissions (a rise of 0.6%) versus 2030-DNS, despite achieving the highest average reduction for all emission types. Similarly, the drop in total emissions for 2030-FE (2%) was much less than the average drop of all emission types (17.2%) against 2030-DNS. This is due to substantial reductions in certain emission types like criteria and toxic pollutants for 2030-I/MP (Section 3.5) and toxic pollutants for 2030-FE (Section 3.6), while almost no reduction was observed for global warming pollutants, which constitute ~94% of daily emissions (Figure 2). This should not discount the effectiveness of 2030-I/MP and 2030-FE since PM_10_ and toxic pollutants have the most damaging effects on health, and, hence, reducing such pollutant loads is expected to result in a reduction in the national health burden [80]. It is important to note that the costs and benefits of each scenario extend beyond emission reduction to include social, environmental, and economic aspects. Hence, a designated study would allow for a more systematic cost–benefit analysis and a proper impact assessment of the five scenarios. Additionally, the 2030 Egypt vision sets a combination of several sustainable transport projects that aim to mitigate emissions, which would be most effective in holistically addressing the issue. Nevertheless, this study evaluated scenarios independently to allow for a clearer basis for comparison.

## 4. Summary, Conclusions, and Future Work

This study estimated the emissions of criteria pollutants (CO, VOC, VOC_evap_, NO_x_, SO_x,_ and PM_10_), toxic pollutants (1, 3 butadien, acetaldehydes, and formaldehydes), and global warming pollutants (CO_2_, N_2_O, and CH_4_) produced by the Greater Cairo vehicle fleet on a given day in 2019. Emissions were then projected from BC-2019 to the year 2030 under 2030-DNS and five emission control scenarios: 2030-FSR, 2030-RE, 2030-PTI, 2030-I/MP, and 2030-FE. The scenarios were chosen based on local agendas and international best practices. The emission amounts for each scenario were contrasted against 2030-DNS to assess their effectiveness. Scenarios were then discussed in comparison to understand the factors pertaining to each scenario. The following key conclusions were drawn.

- CO_2_ emissions constitute 93.7% of total daily on-road transport emission quantities, followed by CO (4.1%) and NO_x_ (1.1%), while the rest constitute ~1% of total emissions. CO_2_ is evidently a problem area that calls for emission control measures to focus on cutting CO_2_ such as 2030-FSR (Section 3.2).
- For the 2019-BC, cars (69% of vehicle fleet) contribute to 35% of daily emissions. Taxis contribute to 24% (despite making up 3% of the fleet), being service vehicles with the second-highest percentage of daily VKT (24%) and number of ‘starts’ (22%). This is also the case for buses (2% of fleet), resulting in 30% of daily emissions. Bus and taxi fleets are also mostly high-age vehicles. Furthermore, 71% and 85% of buses and trucks are diesel-fueled vehicles resulting in 62% and 26% of PM_10_ daily emissions. VKT, vehicle age, and fuel type impact emission quantities. Additionally, results emphasized the prevailing issue of reliance on car-type (cars and taxis) vehicles due to their flexibility and affordability and, hence, call for the introduction of mass transport systems (with the consideration to avoid high-aged and diesel-fueled vehicles).
- The 2030-FSR resulted in an increase in fuel prices, discouraging commutes (8% reduction in VKT) and encouraging the purchase of fuel-efficient and hybrid vehicles. This reduced emissions by, on average, 11.2% in reference to 2030-DNS. The highest reduction in global warming emissions was achieved for cars (12.5%) and motorcycles (12.4%). For 2030-FSR, 16% of cars were replaced by lighter-weight models with improved air/fuel control and hybrid vehicles, while 7% of motorcycles were replaced with models with better air/fuel control. This scenario supports the currently adopted direction of subsidy removal as an effective tool to reduce traffic and associated emissions.
- The benefits of 2030-RE are short-lived, as road expansions create more traffic in the long run. An average increase of 37% in emissions was observed versus 2030-DNS. Trucks cause the highest average percentage increase of emissions (43.6%) since 2030-RE focuses on highways, where trucks travel most. We acknowledge that this control measure would provide improved access to suburban areas, introducing economic benefits, and would create congestion relief in the short term until more sustainable transport projects come online.
- The 2030-PTI provides alternatives to car travel, resulting in reduced car ownership. Consequently, it is estimated to reduce emissions by, on average, 19.5% when referenced to 2030-DNS. Cars result in the most notable reduction for all emission types for 2030-PTI, with an average of 32.8% versus 2030-DNS, which shows how this scenario addresses the core issue of car reliance.
- Enforcing a centralized 2030-I/MP exhibited the largest average reductions in emissions, of 24.4% versus 2030-DNS. Taxis and buses contribute to the most notable average reductions in emissions, of 31.6% and 24.2%, respectively, since a large percentage of aging taxis and buses would be decommissioned in a timelier manner. Global warming emissions exhibited almost no change from 2030-DNS. However, reductions in PM_10_ and toxic pollutants were the most substantial, ranging between 35–54.8%. The 2030-I/MP seems most effective in reducing health-damaging pollutants since they target gross polluters within the vehicle mix. I/M programs are not an evident part of the 2030 Egypt vision; yet, they would be expected to reduce the national health burden and, in turn, economic losses.
- The 2030-FE resulted in an average drop in emissions of 17.2% in reference to 2030-DNS. SO_x_, benzene, and N_2_O emissions were reduced by considerable amounts, of 91.8%, 81%, and 39.1%, respectively. Toxic pollutants were the most reduced for 2030-FE compared to 2030-DNS, where 41% and 47% reductions were observed for cars and trucks, respectively. Global warming pollutants did not seem to benefit from 2030-FE, where minor or no reductions were achieved. Enhancing fuel quality was also not a clear part of the 2030 Egypt vision; nevertheless, our findings highlight its benefits.
- The 2030-FSR has zero capital investment, making its benefit of reducing pollutants attractive. On the other hand, 2030-RE results in a long-term increase in emissions in addition to incurring large economic costs, estimated at US$ 1 million per mile of new lane/road. The 2030-PTI should reduce emissions substantially; however, it requires massive national investments that can reach US$ 300 million for improved public transit. The 2030-I/MP showed the highest reduction in average emissions; however, the cost of an I/M program would vary widely. Hence, it is hard to estimate the funding needed. The 2030-FE costs are also hard to estimate as they are factors of the cost of building refineries locally and the cost of improving vehicle fuel consumption technologies. Access to governmental information is needed to accurately estimate the exact economic costs of implementing each scenario for Greater Cairo.
- The total amount of emissions for each scenario were compared to 2030-DNS, where the highest reduction was observed for 2030-PTI (17.4%), followed by 2030-FSR (11.5%), while 2030-RE resulted in an increase in emissions (37.4%). The 2030-I/MP resulted in almost no change in the total amount of emissions (increase of 0.6%), despite achieving the highest average reduction for all emission types, while the drop in total emissions for 2030-FE (2%) was much less than the average of all emission types (17.2%). This was caused by substantial reductions in certain emission types such as criteria and toxic pollutants for 2030-I/MP and 2030-FE, while almost no reduction was observed for global warming pollutants, which represent a large percentage of total emission quantities. This should not discount the effectiveness of 2030-I/MP and 2030-FE since PM_10_ and toxic pollutants were reported to have more damaging effects on health.

Current interventions to mitigate on-road emissions in Greater Cairo include replacing old-taxis, I/M programs, reducing motorcycle emissions, introducing monorails and BRTs, expanding the metro network, and employing mobile apps for route planning and ride-sharing. This study provides a breakdown assessment for five emissions’ control measures to complement national efforts through assisting policy makers to make informed decisions. We concluded that I/M programs are the most effective in reducing health-damaging pollutants, while improved public transport results in substantial emission reductions in addition to its positive impacts on commuters’ lifestyles. Road expansions, on the other hand, result in higher emissions on the long run. It is worth noting that data availability was the main obstacle in creating the needed database; hence, results are discussed based on the percentage change to allow for applicability. Nevertheless, this study serves as a blueprint for a more detailed study that employs firsthand data. Furthermore, knowledge of expenditure forecasts for each scenario would have allowed for a more accurate cost–benefit analysis to better compare scenarios. This study focused on Greater Cairo, being a worthy representative of growing metropolitan cities in developing countries where environmental considerations need to come hand in hand with economic growth. Overall, this study has put together a preliminary basis for assessing emission control measures that would encourage policy makers and the academic community to utilize such data in making informed decisions for thriving megacities.

## Figures and Tables

**Table 1:** Studies that used modeling tools (including IVE) to assess emissions’ control scenarios in cities similar to Greater Cairo.

Location	Study Focus	Major Findings	Reference
Tehran, Iran	Examining potential actions in mitigating gaseous emissions from vehicles using IVE	Restricting AC usage reduces emissions insignificantly. The idle I/M system can reduce CO and CH_4_ by 10.7 and 3.8%. Sulfur fuel reductions would reduce sulfur emissions by 98%. Substituting old vehicles with new results in reducing CO, VOC, and CH_4_ by 53%, 52%, and 58%.	[40]
Islamabad, Pakistan	Benefits of improved emission control using IVE	CO_2_, CO, NO_x_, SO_2,_ and PM_10_ were reduced for Euro II fuels by 9%, 69%, 73%, 13%, and 31%, while for Euro IV by 5%, 92%, 90%, 92%, and 81%.	[41]
China	NO_x_ emissions from Euro IV buses in urban, suburban, and freeway roads	NO_x_ emissions were more than 2-fold the Euro IV limits for urban, suburban, and freeway driving. NO_x_ emissions were less for vehicles with selective catalytic reduction.	[42]
Delhi, India	Vehicular emission inventory in Delhi using IVE	CO and NOx from personal cars are ~34% and 50% and CO due to two-wheelers is ~61%. Heavy commercial vehicles contribute about 92% of PM.	[43]
Tehran, Iran	On-road vehicle emissions’ forecast using IVE	CO emissions were at 244 tons/hr during peak traffic hours where 25% of this quantity is emitted during start-up.	[44]
Kathmandu Valley, Nepal	Assessing impacts of technologies using IVE	Fleet compliance with Euro III would decrease emissions by 44% for toxic air pollutants and 31% for CO_2eq_.	[45]
Chinese cities	Modeling vehicle emissions using IVE	Euro emission standards could reduce emissions by 30–50%, despite fleet size growth of 15–20% annually.	[46]
Delhi, India	Estimating the total particle number for business-as-usual and best-estimate scenarios	Emissions are expected to increase by 4-times in 2030-business-as-usual but decrease by 18 times in 2030-best-estimate scenarios due to more CNG vehicles and retrofitting of diesel particulate filters.	[47]
Indian cities	Impact of altitude on emissions from light duty vehicles using IVE	Emission rates of CO and VOCs were found to increase with an increase in altitude, with an opposite trend for ambient temperature.	[48]
Hanoi, Vietnam	Emission inventories for motorcycles and light duty vehicles using IVE	Motorcycles contributed most to CO, HC, and NO_x_ emissions while light duty vehicles appeared to consume more fuel.	[49]
Kolkata, India	Assessing the impact of phasing out old vehicles	Higher emissions were observed for non-phasing out of old vehicles compared to phasing-out scenario.	[50]
Chennai, India	Assessing emission control using IVE	Advanced vehicular technology and expansion of public transport resulted in reducing vehicular emissions.	[51]
Wuhan, China	Estimating vehicle emissions at traffic intersections	Simulating bus-driving behavior showed that the simulation model fairly reflects the real driving behavior.	[52]
Beijing and Shanghai, China	Comparison of vehicle activity and emission inventories	3 t of PM, 199 t of NO_x_, 192 t of VOCs, and 2403 t of CO are emitted from on-road vehicles each day in Beijing, whereas 4 t of PM, 189 t of NO_x_, 113 t of VOCs, and 1009 t of CO are emitted in Shanghai.	[53]

## Data Availability

Data is available from the authors on request.

## Supplementary Materials

The following are available online at https://www.mdpi.com/article/10.3390/toxics9110285/s1. Section S1: Vehicle Specific Power (VSP). Figure S1: Greater Cairo road network map. Figure S2: Greater Cairo 2019 vehicle mix. Figure S3: Hourly VKT over 1 day in kilometers for the five vehicle types are shown in the above bar charts for the three road types (a) arterial, (b) highway, and (c) residential. Figure S4: Hourly number of starts over 1 day for the five vehicle types are shown in the above bar charts for the three road types (a) arterial, (b) highway, and (c) residential. Figure S5: Percent distribution of emission quantities for 1 day for 2019 base case. Table S1: Fleet file inputs.
