# Supplementary material for: Emissions Control Scenarios for Transport in Greater Cairo
Source: Toxics. 2021 Nov 1;9(11):285. doi: 10.3390/toxics9110285 (PMC8621196; doi:10.3390/toxics9110285)
Supplement: Supplementary file 1 [file toxics-09-00285-s001.zip › toxics-1397763-SM-cc_RA.pdf]

toxics

MDPI

Supplementary Materials: Emissions Control Scenarios for Transport in Greater Cairo

Rana Alaa Abbas, Prashant Kumar and Ahmed El-Gendy

S1. Vehicle Specific Power (VSP)

Vehicle Specific Power (VSP) and engine stress can be obtained from general knowledge of the vehicle type and a second-by-second velocity trace (ISSRC 2008). The velocity information can be collected on a set of vehicles equipped with combined global positioning satellite units and is then processed in a computer program to estimate VSP and engine stress (ISSRC 2008). The result of the processed data should be the percentage of time spent by the Cairo fleet driving in each of the VSP and stress categories where the IVE tool specifies three stress categories and 20 VSP categories, making a total of 60 bins (ISSRC 2008). Equations S1 and S2 are used to calculate VSP and engine stress.

$$VSP = v[1.1a + 9.81(\text{atan}(\sin(grade))) + 0.132] + 0.000302v^3 \qquad (S1)$$

$$grade = (h_{t=0} - h_{t=-1})/v_{(t=-1to0seconds)}$$

$$v = velocity\left(\frac{m}{s}\right)$$

$$a = acceleration\left(\frac{m}{s^2}\right)$$

$$h = altitude\ (m)$$

$$Engine\ Stress\ (unitless) = RPMIndex + \left(0.08\frac{ton}{kW}\right) * PreaveragePower \qquad (S2)$$

$$PreaveragePower = Average(VSP_{t=-5\,\text{sec}\,to-25sec})\left(\frac{kW}{ton}\right)$$

$$RPMIndex = \frac{Velocity_{t=0}}{SpeedDivider}\ (unitless)$$

Starts have a notable impact on tailpipe emissions where the main effect is the engine soak period before an engine starts (ISSRC 2008). For example, a cold start where the engine has been resting for 18 hours or more would result in the most emissions because the engine must warm up and the catalyst, if one exists, will take longer to heat up to operating conditions (ISSRC 2008). There are ten soak categories within the IVE model, where the soak distribution of how long the engine has been turned off and started, is entered and the entries must add up to 100% (ISSRC 2008). Instruments known as VOCE units are used to collect start patterns data (ISSRC 2008)

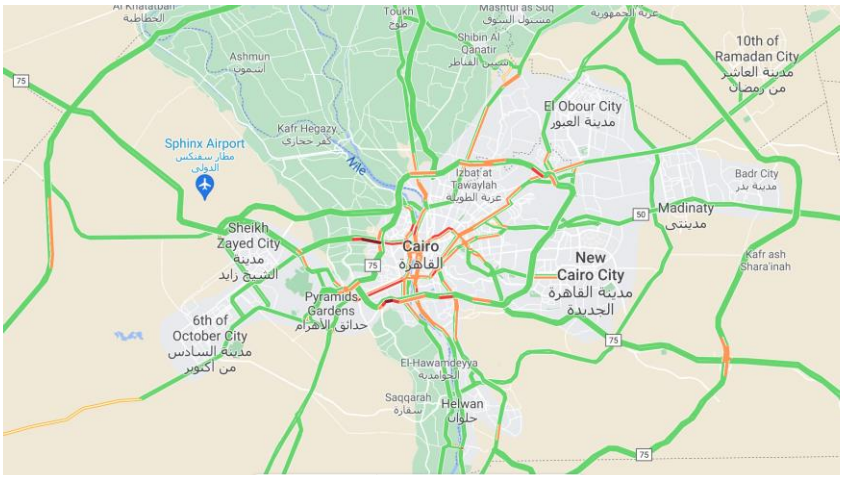

Figure S1. Greater Cairo road network map (Google Maps, 2020).

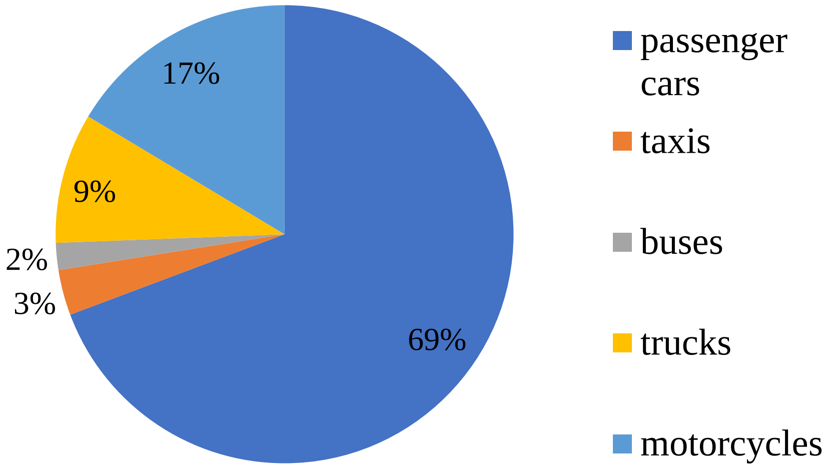

Figure S2. Greater Cairo 2019 vehicle mix (CAPMAS, 2019; CAPMAS 2020).

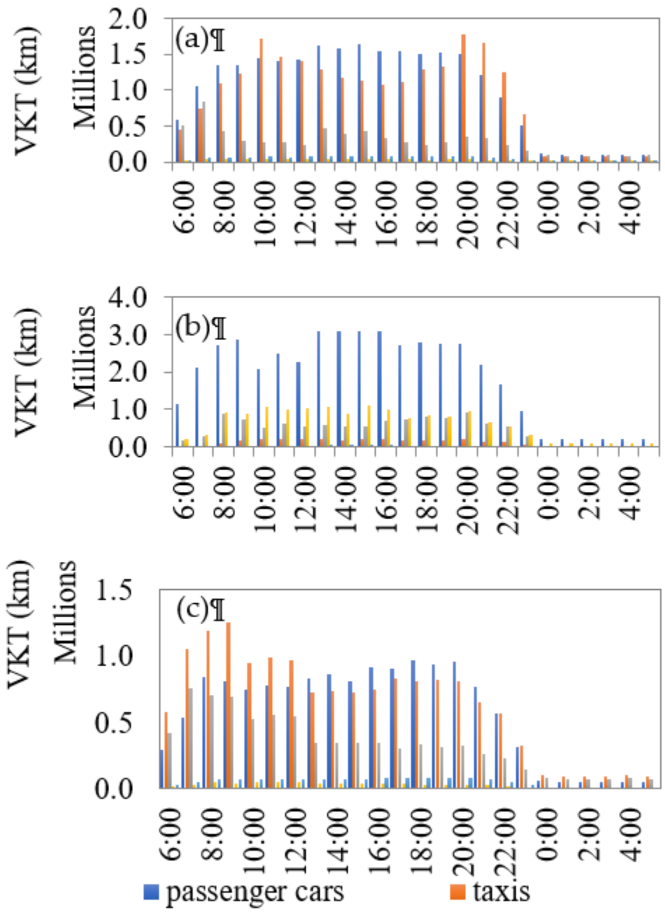

Figure S3. Hourly VKT over one day in kilometers for the five vehicle types are shown in the above bar charts for the three road types (**a**) arterial, (**b**) highway and (**c**) residential.

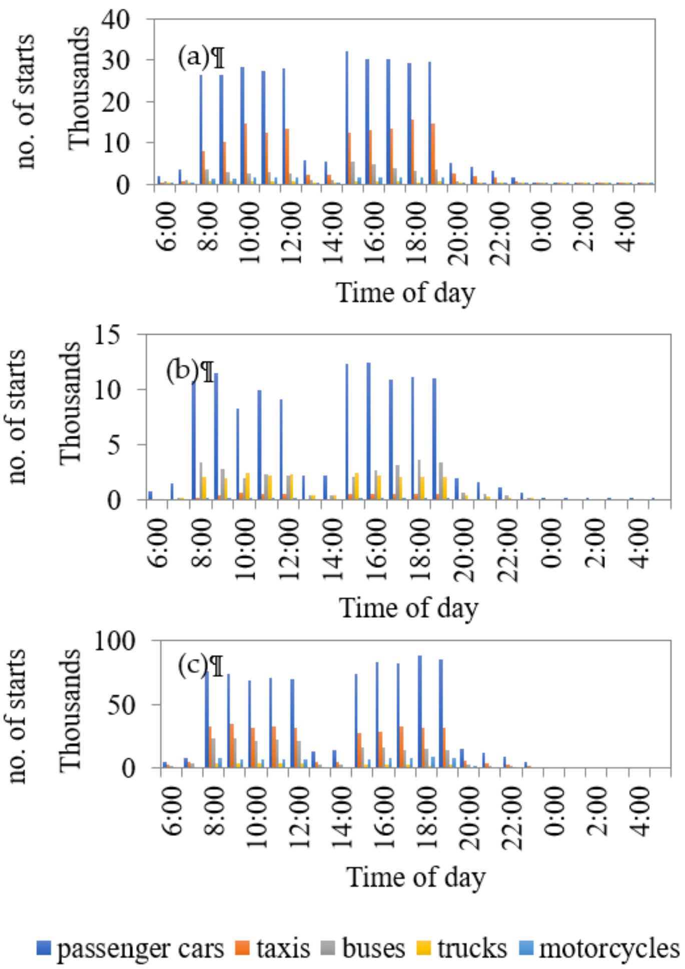

Figure S4. Hourly number of starts over one day for the five vehicle types are shown in the above bar charts for the three road types (a) arterial, (b) highway and (c) residential.

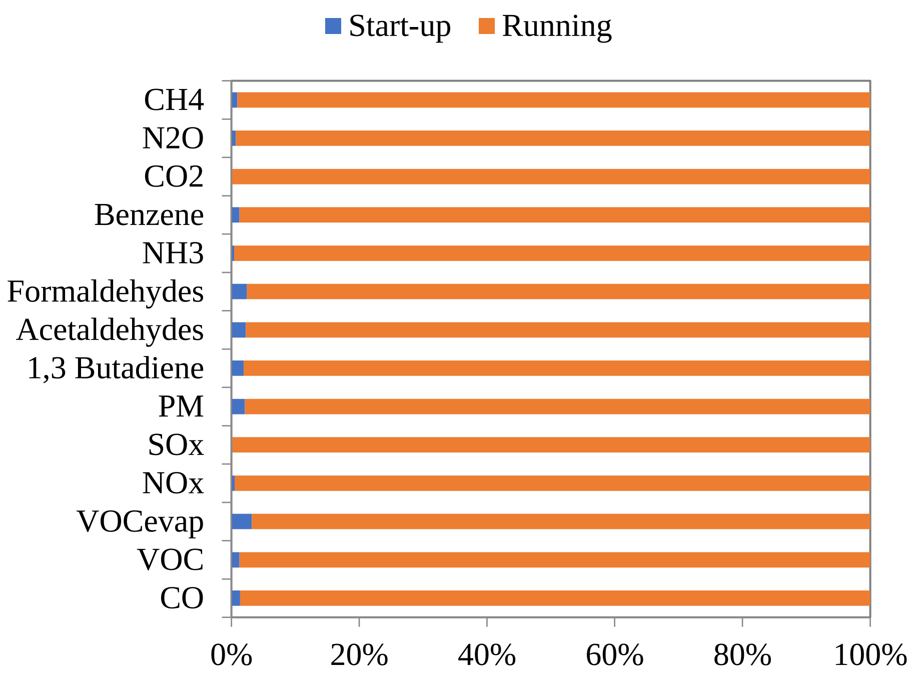

Figure S5. Percent distribution of emission quantities for one day for 2019 base case.

Table S1. Fleet file inputs.

Description	Fuel	Weight	Air/Fuel Control	Exhaust	Evaporative	Age	Index	%
				Passenger cars				
Auto/Sml Truck	Petrol	Light	Carburetor	2-Way	PCV	>161K km	11	8.2
Auto/Sml Truck	Petrol	Medium	Carburetor	2-Way	PCV	>161K km	14	14.2
Auto/Sml Truck	Petrol	Heavy	Carburetor	2-Way	PCV	>161K km	17	0.5
Auto/Sml Truck	Petrol	Light	Single-Pt FI	3-Way	PCV	80–161K km	82	11.2
Auto/Sml Truck	Petrol	Medium	Single-Pt FI	3-Way	PCV	80–161K km	85	19.3
Auto/Sml Truck	Petrol	Heavy	Single-Pt FI	3-Way	PCV	80–161K km	88	0.6
Auto/Sml Truck	Petrol	Light	Multi-Pt FI	EuroIII	PCV/Tank	<79K km	189	7.2
Auto/Sml Truck	Petrol	Medium	Multi-Pt FI	EuroIII	PCV/Tank	<79K km	192	12.4
Auto/Sml Truck	Petrol	Heavy	Multi-Pt FI	EuroIII	PCV/Tank	<79K km	195	0.4
Auto/Sml Truck	Natural Gas	Light	Carb/Mixer	2-Way	PCV	>161K km	272	1.4
Auto/Sml Truck	Natural Gas	Medium	Carb/Mixer	2-Way	PCV	>161K km	275	2.4
Auto/Sml Truck	Natural Gas	Heavy	Carb/Mixer	2-Way	PCV	>161K km	278	0.1
Auto/Sml Truck	Natural Gas	Light	FI	3-Way	PCV	<79K km	306	1.2
Auto/Sml Truck	Natural Gas	Light	FI	3-Way	PCV	80–161K km	307	1.9
Auto/Sml Truck	Natural Gas	Medium	FI	3-Way	PCV	<79K km	309	2.1
Auto/Sml Truck	Natural Gas	Medium	FI	3-Way	PCV	80–161K km	310	3.2
Auto/Sml Truck	Natural Gas	Heavy	FI	3-Way	PCV	<79K km	312	0.1
Auto/Sml Truck	Natural Gas	Heavy	FI	3-Way	PCV	80–161K km	313	0.1
Auto/Sml Truck	Diesel	Light	Pre-Chamber Inject.	Improved	None	>161K km	749	1.5
Auto/Sml Truck	Diesel	Medium	Pre-Chamber Inject.	Improved	None	>161K km	752	2.6
Auto/Sml Truck	Diesel	Heavy	Pre-Chamber Inject.	Improved	None	>161K km	755	0.1
Auto/Sml Truck	Diesel	Light	Direct Injection	EGR+Improv	None	<79K km	756	1.3
Auto/Sml Truck	Diesel	Medium	Direct Injection	EGR+Improv	None	<79K km	759	2.2
Auto/Sml Truck	Diesel	Heavy	Direct Injection	EGR+Improv	None	<79K km	762	0.1
Auto/Sml Truck	Diesel	Light	FI	EuroIII	None	80–161K km	802	2.1
Auto/Sml Truck	Diesel	Medium	FI	EuroIII	None	80-161K km	805	3.6
Auto/Sml Truck	Diesel	Heavy	FI	EuroIII	None	80-161K km	808	0.1
				Taxis				
Auto/Sml Truck	Petrol	Light	Carburetor	2-Way	PCV	>161K km	11	8.1
Auto/Sml Truck	Petrol	Medium	Carburetor	2-Way	PCV	>161K km	14	14.0
Auto/Sml Truck	Petrol	Heavy	Carburetor	2-Way	PCV	>161K km	17	0.5
Auto/Sml Truck	Petrol	Light	Single-Pt FI	3-Way	PCV	80–161K km	82	4.7
Auto/Sml Truck	Petrol	Medium	Single-Pt FI	3-Way	PCV	80–161K km	85	8.2
Auto/Sml Truck	Petrol	Heavy	Single-Pt FI	3-Way	PCV	80–161K km	88	0.3
Auto/Sml Truck	Petrol	Light	Multi-Pt FI	EuroIII	PCV/Tank	<79K km	189	0.7
Auto/Sml Truck	Petrol	Medium	Multi-Pt FI	EuroIII	PCV/Tank	<79K km	192	1.2
Auto/Sml Truck	Petrol	Heavy	Multi-Pt FI	EuroIII	PCV/Tank	<79K km	195	0.0
Auto/Sml Truck	Natural Gas	Light	Carb/Mixer	2-Way	PCV	>161K km	272	3.4
Auto/Sml Truck	Natural Gas	Medium	Carb/Mixer	2-Way	PCV	>161K km	275	5.9
Auto/Sml Truck	Natural Gas	Heavy	Carb/Mixer	2-Way	PCV	>161K km	278	0.2
Auto/Sml Truck	Natural Gas	Light	FI	3-Way	PCV	<79K km	306	0.3
Auto/Sml Truck	Natural Gas	Light	FI	3-Way	PCV	80–161K km	307	2.0
Auto/Sml Truck	Natural Gas	Medium	FI	3-Way	PCV	<79K km	309	0.5
Auto/Sml Truck	Natural Gas	Medium	FI	3-Way	PCV	80–161K km	310	3.5
Auto/Sml Truck	Natural Gas	Heavy	FI	3-Way	PCV	<79K km	312	0.0
Auto/Sml Truck	Natural Gas	Heavy	FI	3-Way	PCV	80–161K km	313	0.1
Auto/Sml Truck	Diesel	Light	Pre-Chamber Inject.	Improved	None	>161K km	749	10.0
Auto/Sml Truck	Diesel	Medium	Pre-Chamber Inject.	Improved	None	>161K km	752	17.3

Auto/Sml Truck	Diesel	Heavy	Pre-Chamber Inject.	Improved	None	>161K km	755	0.6
Auto/Sml Truck	Diesel	Light	Direct Injection	EGR+Improv	None	<79K km	756	0.8
Auto/Sml Truck	Diesel	Medium	Direct Injection	EGR+Improv	None	<79K km	759	1.4
Auto/Sml Truck	Diesel	Heavy	Direct Injection	EGR+Improv	None	<79K km	762	0.1
Auto/Sml Truck	Diesel	Light	FI	EuroIII	None	80–161K km	802	5.9
Auto/Sml Truck	Diesel	Medium	FI	EuroIII	None	80–161K km	805	10.1
Auto/Sml Truck	Diesel	Heavy	FI	EuroIII	None	80–161K km	808	0.3
Buses								
Truck/Bus	Petrol	Medium	Carburetor	2-Way	PCV	>161K km	842	5.5
Truck/Bus	Petrol	Heavy	Carburetor	2-Way	PCV	>161K km	845	4.3
Truck/Bus	Petrol	Medium	FI	EuroII	PCV	80–161K km	931	3.2
Truck/Bus	Petrol	Heavy	FI	EuroII	PCV	80–161K km	934	2.5
Truck/Bus	Petrol	Medium	FI	EuroIII	PCV	<79K km	939	0.5
Truck/Bus	Petrol	Heavy	FI	EuroIII	PCV	<79K km	942	0.4
Truck/Bus	Natural Gas	Medium	Carb/Mixer	None	PCV	<79K km	966	0.4
Truck/Bus	Natural Gas	Medium	Carb/Mixer	None	PCV	80–161K km	967	2.8
Truck/Bus	Natural Gas	Medium	Carb/Mixer	None	PCV	>161K km	968	4.7
Truck/Bus	Natural Gas	Heavy	Carb/Mixer	None	PCV	<79K km	969	0.2
Truck/Bus	Natural Gas	Heavy	Carb/Mixer	None	PCV	80–161K km	970	1.7
Truck/Bus	Natural Gas	Heavy	Carb/Mixer	None	PCV	>161K km	971	2.9
Truck/Bus	Diesel	Medium	Pre-Chamber Inject.	None	None	>161K km	1076	18.4
Truck/Bus	Diesel	Heavy	Pre-Chamber Inject.	None	None	>161K km	1079	24.3
Truck/Bus	Diesel	Medium	FI	EuroII	None	80–161K km	1129	10.7
Truck/Bus	Diesel	Heavy	FI	EuroII	None	80–161K km	1132	14.2
Truck/Bus	Diesel	Medium	FI	EuroIII	None	<79K km	1137	1.5
Truck/Bus	Diesel	Heavy	FI	EuroIII	None	<79K km	1140	2.0
Trucks								
Truck/Bus	Petrol	Light	Carburetor	None	PCV	>161K km	830	7.1
Truck/Bus	Petrol	Medium	Carburetor	None	PCV	>161K km	833	2.5
Truck/Bus	Petrol	Heavy	Carburetor	None	PCV	>161K km	836	4.7
Truck/Bus	Petrol	Light	FI	EuroI	PCV	80–161K km	919	2.0
Truck/Bus	Petrol	Medium	FI	EuroI	PCV	80–161K km	922	0.7
Truck/Bus	Petrol	Heavy	FI	EuroI	PCV	80–161K km	925	1.3
Truck/Bus	Petrol	Light	FI	EuroII	PCV	<79K km	927	1.0
Truck/Bus	Petrol	Medium	FI	EuroII	PCV	<79K km	930	0.4
Truck/Bus	Petrol	Heavy	FI	EuroII	PCV	<79K km	933	0.7
Truck/Bus	Diesel	Light	Pre-Chamber Inject.	None	None	>161K km	1073	40.0
Truck/Bus	Diesel	Medium	Pre-Chamber Inject.	None	None	>161K km	1076	0.1
Truck/Bus	Diesel	Heavy	Pre-Chamber Inject.	None	None	>161K km	1079	15.5
Truck/Bus	Diesel	Light	Direct Injection	Improved	None	<79K km	1080	5.7
Truck/Bus	Diesel	Medium	Direct Injection	Improved	None	<79K km	1083	0.0
Truck/Bus	Diesel	Heavy	Direct Injection	Improved	None	<79K km	1086	2.2
Truck/Bus	Diesel	Light	FI	EuroI	None	80–161K km	1117	11.4
Truck/Bus	Diesel	Medium	FI	EuroI	None	80–161K km	1120	0.0
Truck/Bus	Diesel	Heavy	FI	EuroI	None	80–161K km	1123	4.4
Motorcycles								
Sml Engine	Petrol	Med	4-Cycle, Carb	None	None	0–25K	1209	5
Sml Engine	Petrol	Med	4-Cycle, Carb	None	None	26–50K	1210	25
Sml Engine	Petrol	Med	4-Cycle, Carb	None	None	>50K	1211	70
